# Internal Biomechanical Study of a 70-Year-Old Female Human Lumbar Bi-Segment Finite Element Model and Comparison with a Middle-Aged Male Model

**DOI:** 10.1155/2019/9794365

**Published:** 2019-04-30

**Authors:** Hequan Wu, Jinping Peng, Xin Jin

**Affiliations:** ^1^Key Laboratory of Lightweight and Reliability Technology for Engineering Vehicle, Education Department of Hunan Province, Changsha University of Science and Technology, Hunan, Changsha 410004, China; ^2^Bioengineering Center, Wayne State University, Detroit, MI 48 202, USA

## Abstract

The main purpose of this article is to study the biomechanics of spine tissue in elderly female. In this study, the L3-L5 lumbar bi-segmental finite element model for elderly female was obtained from the Advanced Human Modeling Laboratory of the Bioengineering Center at Wayne State University. The effects of flexion and extension on bone geometry, distribution of ligament fibers, location of nucleus, and changes in intervertebral disc height were studied by comparing the results obtained before and after the update of older female and middle-aged male models. For the purpose of comparing the calculated range of motion (ROM) with the experimental data, additional calculations for axial rotation and lateral bending were performed. The study found that the parameters of the model affected the deformation of the disc herniation, ligament and intervertebral disc, and the axial force carrying capacity of the model. The three predicted ROMs are usually similar to the experimental results. Only the older female model has a slightly larger ROM. Therefore, older women are more vulnerable to lumbar spine injuries than men.

## 1. Introduction

Over the past 60 years, a large number of cadaveric tests have been conducted in order to study the lumbar biomechanics under conditions of health and pathology [1–3]. In these tests, the force analysis of different lumbar components under different loading conditions was studied to evaluate the biomechanical properties of the lumbar spine and the components of the lumbar spine. However, there are great changes in the experiments themselves. As the differences in bodies of each test, as well as deviations in the presence of the test, etc., the geometric or tissue state of the lumbar spine cannot be determined [4–6].

As long as a thorough validation process is performed, analysis of the spine will have a positive effect on future experiments. Unfortunately, many published models have not been validated [7–13]. Among them, some reports only performed a comparison of ROM and intervertebral disc pressure with experimental results, or the study of material properties [7, 14–19].

In the light of U.S. Census Bureau data reported by the National Highway Traffic Safety Administration, the population aged 65 and over will increase by nearly 10% in the next few decades. The lumbar vertebrae are a relatively vulnerable part of the elderly population, so the research of the lumbar spine biomechanics of the elderly has become more important, especially for older women.

The primary objective of this study was to evaluate the interaction between the model stress distribution and the range of lumbar motion. Even if the geometric model is not a determinant from the perspective of the resulting range of motion (ROM), it may have a great influence on the relative effects of different model components. Comparing the existing male model [20], the new middle-aged male model [21], and the Wayne State University's older female model, it is assumed that all three models are capable of predicting similar movements and can be verified by comparing the extracorporeal segment motion data.

## 2. Methods

**Figure 1:**
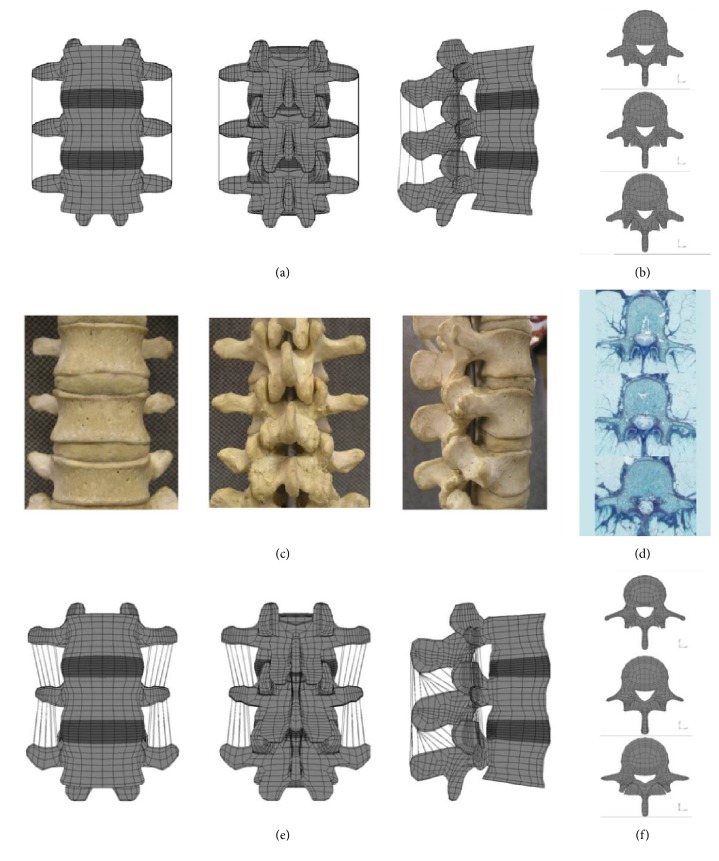
L3–L5 lumbar spine bi-segment three-way view and upper view of each vertebra. (a, b) Old model, (c, d) natural bone and (e, f) new model.

Noailly et al. [8] modified the L3-L5 bi-segment finite element model created in the smit [20] study for all lumbar spine components. In the model created by smit, the L4 vertebra was from a computed tomography scan of a middle-aged male, and L3 and L5 were replicated from L4. Thus, although Noailly et al. [8] updated the model, there was no significant difference in the motion of the L3-L4 and L4-L5 segments. So this old model (Figures 1(a) and 1(b)) was used as a reference and a new, more accurate geometric model was created to study the characteristics of the lumbar spine. The L3-L5 vertebrae were developed based on the relative size and shape of the matched set of preexisting L4 model and a group of male spinal corpses. Whereas the length, width, and depth of the L3 and L5 cones of the new geometric model (Figures 1(e) and 1(f)) are still proportional to L4, and the proportional ratios are calculated from the average quantitative geometric data given by Panjabi et al. [22]. They were then subjected to flexion and extension tests to determine the relative biomechanical effects of middle-aged spine tissue.

**Figure 2:**
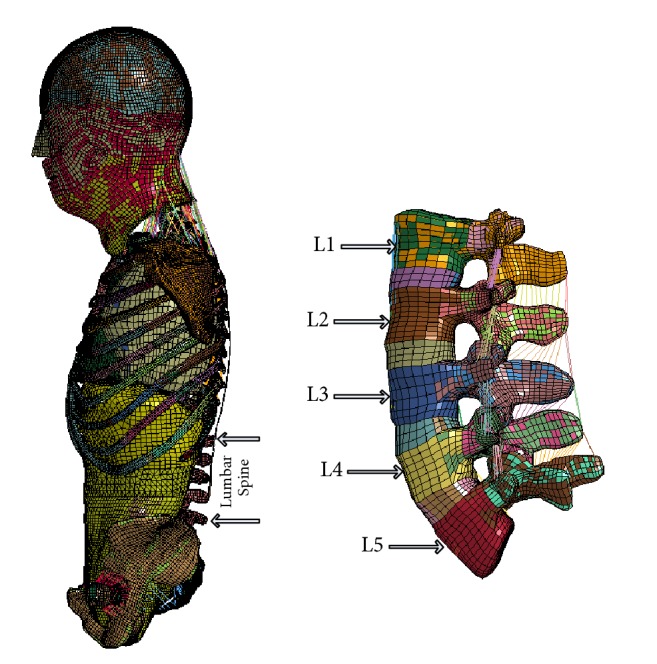
Lumbar position in the human body.

The lumbar spine is part of the spine and supports the upper body weight. The lumbar spine has a large range of motion in the overall movement of the spine and can complete the rotation, flexion, and lateral flexion of the trunk. As part of the posterior wall of the abdomen, it participates in the abdominal cavity. The part of the lumbar spine in the human body is shown in Figure 2.

**Figure 3:**
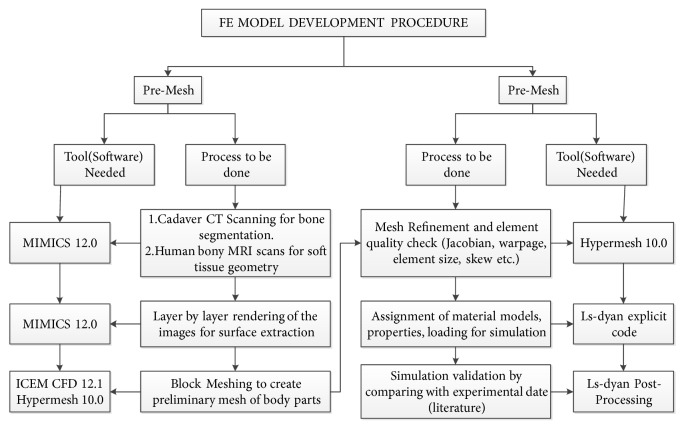
A schematic diagram for the development of an FE human model.

In this study, the previous old model and the new model were only used as references for studying the biomechanical properties of lumbar spine in elderly female. We need a lumbar model that represents 70-year-old women. The establishment of elderly female spine models is complex. Researchers in the Advanced Human Modeling Laboratory of the Bioengineering Center at Wayne State University continue to develop numerical models to analyze the effects of car crashes on human body. The FE model can be developed by using modeling software such as Mimics (Materialise, Leuven, Belgium), Hypermesh (Altair Engineering Inc., Troy, MI, USA), and LS-DYNA (LSTC, Livermore, CA, USA). The schematic diagram for the development of a human model is shown in Figure 3. The same approach was applied in this research for the development of an FE model representing a 70-year-old female. In addition, the current study also employed statistical data to ensure that the eventual FE model represents an average 70-year-old female.

**Figure 4:**
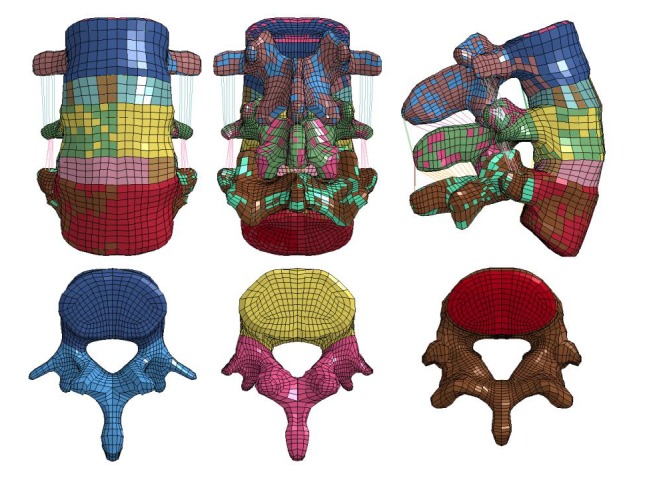
The L3-L5 lumbar spine bi-segment three-way view of the elderly female model and the upper view of each vertebra.

As previously described, an average 70-year-old female has anthropometric measurements of 1.6 m in height and 73 kg in weight, based on statistical results from Centers for Disease Control and Prevention. But, it is not an easy task to find a cadaver which matches exactly the same anthropomorphic details as described through Centers for Disease Control and Prevention data. Therefore, the closest match—a female cadaver (73 years old) with a height of 1.6 m and a weight of 62 kg—was selected to extract skeleton geometry from computed tomography scan images with the approval of the Institutional Review Board/Human Investigation Committee of Wayne State University. The cadaver was scanned at the Oakwood Hospital Radiology Department in Taylor, MI. The old female lumbar model required for the experiment was extracted (Figure 4).

Noailly et al. [21] have an introduction to the material properties of male old model and new model. The material properties of the older female model were taken from the Advanced Human Modeling Laboratory at the Wayne State University Bioengineering Center (Table 1). The purpose of this paper is to study the most commonly used constitutive relationships in elderly women's lumbar models by comparing them with middle-aged male models. Only the kinematics of the lumbar spine under static conditions were studied, and the loss of energy was not considered. An axial, sagittal, and frontal pure rotational moment of 7.5 Nm is applied at the top of L3 and all degrees of freedom of the lower half of L5 are constrained. In the simulation, the load is set to a single pure moment centered on the horizontal plane of the L3 upper end plate. They act on the two sides through the shell elements and stick to the surrounding end plate hexahedral elements through the contact process. The boundary conditions of this experiment were determined according to a custom spine tester developed by Wilke et al. [23] and the results of the ROM were compared. Under sagittal motion, the spine model determined the effect on the spine by continuously removing the ligament (Table 2). The ROM and surface contact force were calculated, and the relative differences between the old model, the new model, and the older female model were analyzed. Then the deformed figure of the aged female model under flexion and stretching was drawn, and the change of the aged female model was analyzed.

The steps used are all done on LS-dyan.

## 3. Results

### 3.1. Flexion

**Figure 5:**
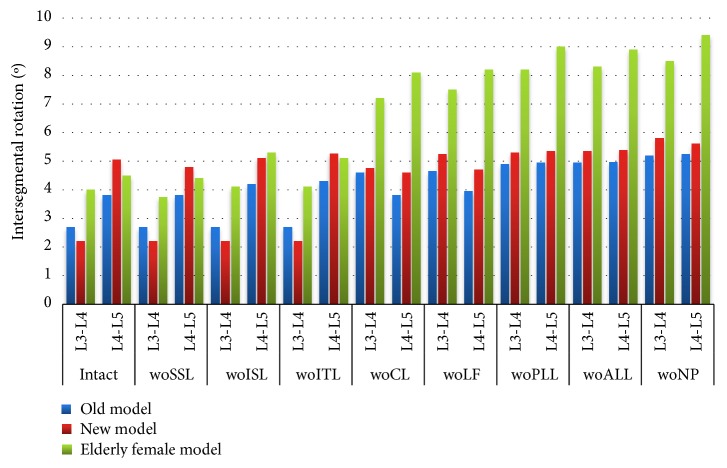
Under the sagittal flexion of 7.5 Nm, the absolute value of the range of motion of the intact model and after each component resection. Abbreviation Vocabulary Reference Table 2.

During flexion, the ROM of the lumbar spine increases with the removal of each component (Figure 5). The ROM of the L3-L4 segment and L4-L5 segment of the elderly female model significantly increased after the removal of the capsular ligament (CL). In the new male model and the old male model (hereinafter referred to as the new model and the old model), the L3-L4 segment is usually stiffer than the L4-L5 segment until the transverse ligament (ITL) is removed. Until the intertransverse ligament (ITL) is removed, the L3-L4 segments of the three models are usually stiffer than the L4-L5 segments. In the female model, the stiffness of the L3-L4 segment and that of the L4-L5 segment are similar. The lumbar motion range of a female model is usually larger than the old and the new model. As the age of older women increases, the degree of deformation of each segment increases and the hardness of bones decreases.

**Figure 6:**
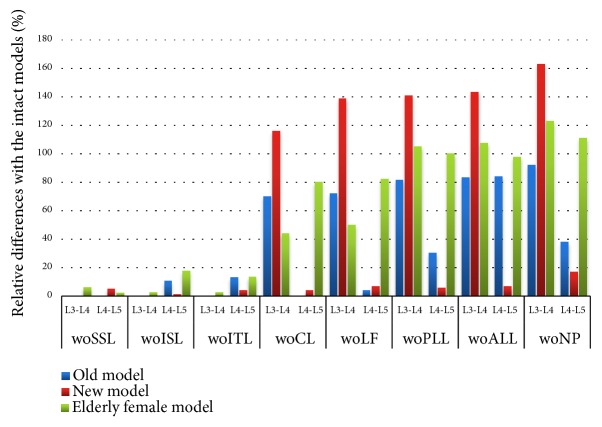
Under the sagittal flexion of 7.5 Nm, the relative difference in range of motion after each component resection. Abbreviation Vocabulary Reference Table 2.

Before the removal of the capsular ligament (CL), the ROM of the old model, the new model, and the L3-L4 segment of the older female model did not change significantly (Figure 6), but after removal, the sensitivity increased. The rotational stiffness of the L3-L4 segment was reduced by approximately 50% in the elderly female model after removal of the capsular ligament (CL), and the rotational stiffness was reduced by approximately 105% after removal of the posterior longitudinal ligament (PLL) and anterior longitudinal ligament (ALL). The SSL removal reduces the rotational stiffness of the L4-L5 segment of the new model by approximately 4%, and the CL removal reduced the L3-L4 rotational stiffness by approximately 20%. In the elderly female model, the rotational stiffness of L4-L5 segments gradually decreases with the removal of various components.

**Figure 7:**
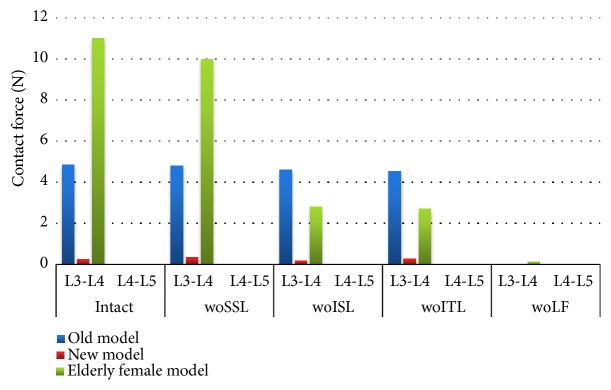
Under the sagittal flexion of 7.5 Nm, the normal facet contact forces of the intact model and after each component resection. Abbreviation Vocabulary Reference Table 2.

In the three models, the normal facet contact force only exists in the L3-L4 segment (Figure 7). Initially, the contact force of the old model was 20 times higher than the new model, and the contact force of the female model is nearly 3 times larger than the new model. Once the LF is removed, there is almost no contact force in models. In the new model, the removal of SSL and ISL resulted in a 70% increase in L3-L4 contact force and a 20% decrease in L3-L4 contact force, respectively. In elderly female model, removal of ISL resulted in a 70% reduction in contact force.

**Figure 8:**
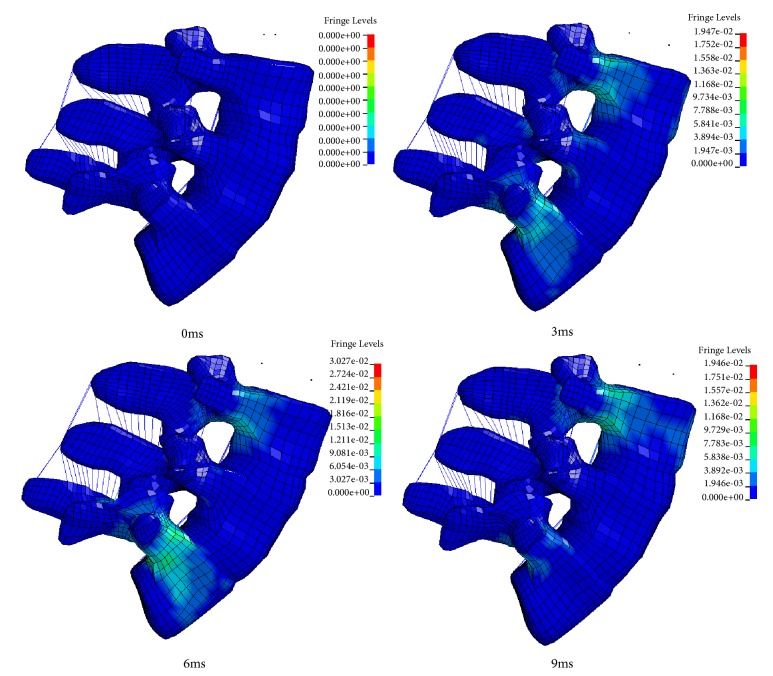
Stress cloud image under an intact elderly female model under 7.5 Nm sagittal flexion. The stress unit is Gpa.

Figure 8 shows the cross-sectional deformation and stress plots of the lumbar L3-L5 segments within a 3ms interval in the simulation. Under the sagittal flexion of 7.5 Nm, range of motion of the lumbar spine increased and the maximum stress occurs at the vertebral junction between the spine and L5.

### 3.2. Extension

**Figure 9:**
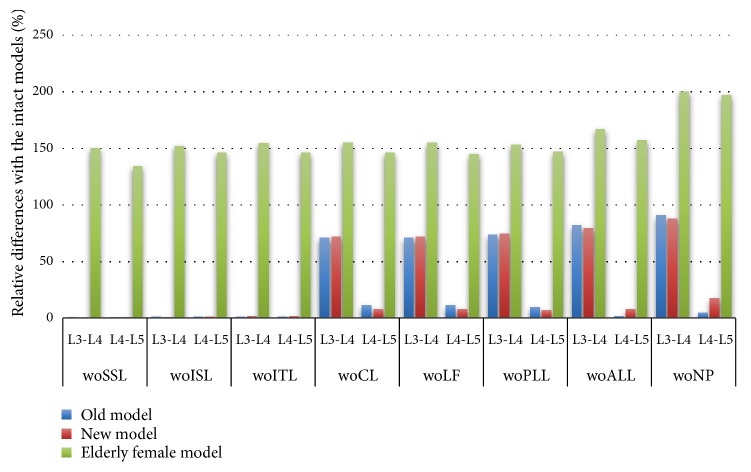
Under the sagittal extension of 7.5 Nm, the relative difference in range of motion after each component resection. Abbreviation Vocabulary Reference Table 2.

In extension, the effect of removing CL from the old model and the new model is similar to that of buckling (Figure 9). However, in the elderly female model, the sensitivity after SSL removal is very large, and the rotation stiffness of L3-L4 segment and L4-L5 segment is greatly reduced.

**Figure 10:**
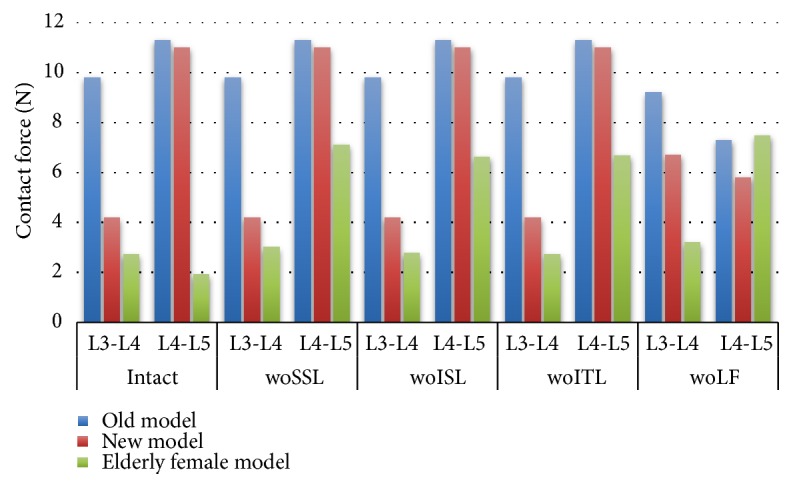
Under the sagittal extension of 7.5 Nm, the normal facet contact forces of the intact model and after each component resection. Abbreviation Vocabulary Reference Table 2.

The L3-L4 predictive contact force value of the intact model in the old model is more than twice that of the new model, which is more than three times higher than that of the older female model (Figure 10). ISL suppression only helps to reduce the contact force of the new model. LF removal helps to reduce the contact force of the elderly female model L3-L4 level. The relative difference in contact force indicates that, at the L3-L4 level, the contact force changes of the new model and the older female model are nearly 10 times higher than the old model. In L4-L5, the contact force change in the new model is about 13% higher than that of the old model, and the older female model is about 2 times higher than the new model.

**Figure 11:**
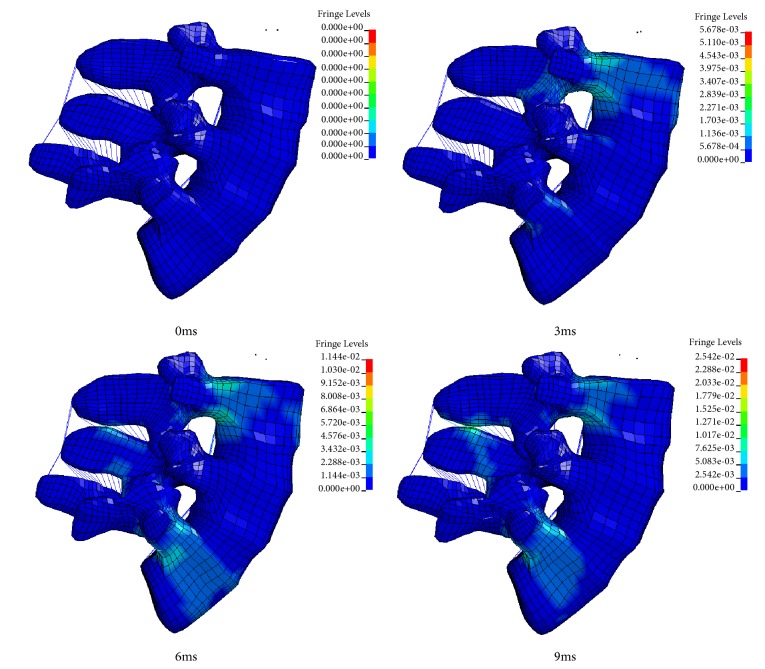
Stress cloud image of the complete female model under 7.5 Nm sagittal flexion. The stress unit is Gpa.

Under a 7.5Nm torque sagittal plane stretching, 3 ms was taken as an interval to observe the deformation and stress cloud of the lumbar L3-L5 segment (Figure 11). As time increases, the deformation increases and the stress on the lumbar spine increases. The main stress occurs at the junction of the spine and the spine with the vertebral body.

### 3.3. Comparison with Experimental Results

**Figure 12:**
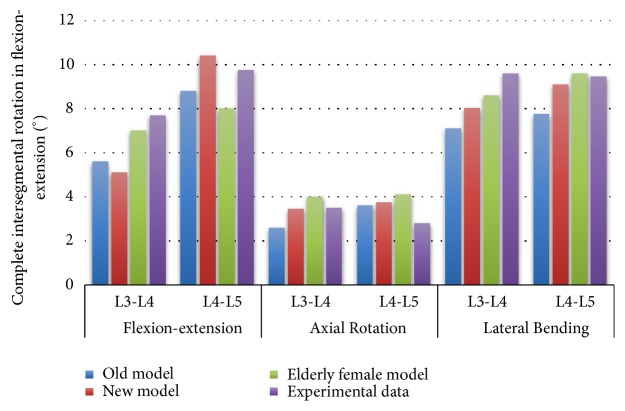
Comparison of the range of motion between experiments and simulations when applying a pure torque of 7.5 Nm under a complete model.

ROM data on cadaveric experiments and three models showed that the three-middle model matched the experimental results, and the ROM of the older female model was slightly larger than the old and new models (Figure 12). In the experiment of Bell et al. [24], the total range of motion of the selected female spine specimens was 10.7 ± 0.6, 4.8 ± 0.6, and 10.2 ± 1.5 (mean ± SD) degrees of flexion/extension, axial rotation, and lateral bending. This also matches well with this model.

## 4. Discussion

After the CL is removed, the ROM of the lumbar spine changes very significantly. This prediction is the same as some of the experiments previously reported (Adams et al., 1980; [21, 25]). The results show that CL plays a significant role in inhibiting the movement of the lumbar spine during flexion. In the simulation of stretching of the older female model, the removal of SSL plays a key role in the ROM of the lumbar spine. Whatever the model, whether or not the BPE is removed has no significant effect on the ROM at the time of flexion, which is similar to previous studies (Schultz et al., 1979).

In the L3-L4 segment, the components of the restricted model ROM are not only LF, but also the PLL in the older female model and the PLL in the old model. In flexion, the L3-L4 level is different from the inhibition model component in the L4-L5 level. In the elderly female model, ISL, LF, and PLL all play a role in suppressing the deformation of the model. The removal of CL and PLL increases the range of motion of the lumbar spine of the old and new models to some extent.

In the process of extension deformation, the deformation of old female model is not the same as the deformation of new model and old model. It is SSL that plays a major role in suppressing the distortion of older female models. For the old model and the new model, CL plays a role in suppressing the deformation of the model. The removal of ALL reduced the deformation of the L4-L5 segment of the old model.

By comparing the flexion and extension deformation between old model, new model, and older female model, it is found that the deformation of the L3-L4 and L4-L5 segments of the lumbar vertebra in older female model is generally higher than the old model and new model. This is due to the fact that, in the age of the elderly, bones and ligaments have a certain degree of rigidity. After the removal of a specific ligament, such as LF, the ROM of older female models is larger than that of old and new models.

Comparing the normal facet contact force of corresponding segments of three models, in flexion, the contact force of the L3-L4 segment decreases as the components are removed. In extension, the new model and the older female model have the same trend in the L3-L4 segment, and there was no significant change in the old model. After the SSL removal, the contact force of the L4-L5 level of the elderly female model increased.

From the stress cloud of the elderly female model, it can be seen that the stress concentration mainly occurs in the third lumbar vertebral body, the fifth lumbar vertebral body, and the vertebrae connected with the flexion and extension exercises. The maximum stress occurs at the junction of the spine and the vertebral body. It may indicate that the lumbar ligament has certain limitations on the movement of the lumbar spine.

It seems that despite the older female model, the old and new model simulation, and experimental ROM, the ligament fiber orientation and distribution, the joint direction, the NP position, etc. have matching rotation angles. However, each model has specific interactions, so their respective biomechanical behaviors are very different. Therefore, although the older female models seem to have more physiological characteristics than the old and new models, there is no real conclusion to illustrate the better effectiveness of one of the construction models. Therefore, verification by calculating the comparison between global characteristics and experimental results does not guarantee the correlation between them.

However, it can be seen from the experimental and simulation data that the lumbar model of the elderly female matches the bending angles of the L3-L4 and the L4-L5 segments during flexion/extension, axial rotation, and lateral bending. Therefore, there is a certain guarantee for the accuracy of the old female model.

## 5. Conclusion

From the comparison of the range of lumbar motion between the elderly female model and the middle-aged male, it can be seen that the elderly female model has good fidelity.

From the above discussion, it can be seen that the stiffness of the lumbar vertebrae, ligaments, and intervertebral discs of the older female model is smaller than that of the old and new models. Therefore, the lumbar vertebrae stiffness of older women should be slightly smaller than the lumbar vertebrae stiffness of middle-aged men. When affected by the same impact, the lumbar vertebrae of elderly women suffer more damage.

However, due to limited experimental data and models, this experiment only performed partial experiments on a single lumbar vertebra, but no global experiments were performed. This model can only represent the biomechanical properties of 70-year-old elderly women. In order to obtain more accurate lumbar spine biomechanics for older women of all ages, more experiments are needed to compare.

## Figures and Tables

**Table 1:** Summary of material properties for modeling, all material attributes are from Wayne State University(Where Ro-Mass density, E-Young's modulus,PR-Poisson's ratio).

Material	Ro (kg/mm^3^)	E (GPa)	PR
Trabecular bone	1.1000e-6	0.200000	0.200000

Cortical bone	1.7000e-6	15.000000	0.300000

Bony endplate	1.5000e-6	0.023800	0.400000

Vertebral	1.4000e-6	3.500000	0.250000

Ligaments	1.0000e-6	0.0	—* *—

Annulus fibrosus fibres	1.0000e-6	0.023375	0.3500000

Nucleus pulposus	1.0200e-6	0.0010000	0.4999000

**Table 2:** Description of the stepwise resection of the spine component on the model.

Model	woSSL	woISL	woITL	woCL	woLF	woPLL	woALL	woNP
Removed component	Supraspinous ligament	Interspinous ligament	Intertransverse ligament	Capsular ligament	Ligamentum Flavum	Posterior longitudinal ligament	Anterior longitudinal ligament	Nucleus pulposus
	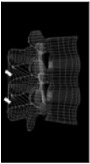	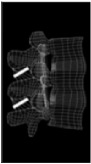	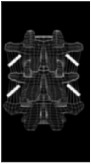	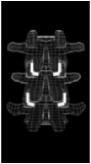	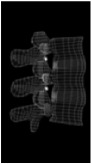	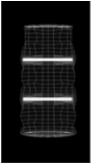	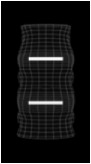	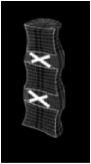

## Data Availability

In this study, the accurate L3-L5 lumbar bi-segmental finite-element model for elderly female was obtained from the Advanced Human Modeling Laboratory of the Bioengineering Center at Wayne State University. Under the sagittal flexion of 7.5 Nm, when the step-by-step NP (nucleus pulposus) is also removed, we can see that the range of motion for L3-L4 segments is 8.5° and the range of motion for L4-L5 segments is 9.4°. The specific model refers to spine.k.
